# Pre-discharge energy intake and post-discharge mortality in acutely hospitalized older adults

**DOI:** 10.1007/s40520-025-03076-7

**Published:** 2025-05-26

**Authors:** Tomihiko Tajima, Hirotaka Nakashima, Masaaki Nagae, Hitoshi Komiya, Chisato Fujisawa, Kazuhisa Watanabe, Yosuke Yamada, Hiroyuki Umegaki

**Affiliations:** 1 Department of Community Healthcare and Geriatrics, Nagoya University Graduate School of Medicine, 65 Tsurumai-cho, Showa-ku, Nagoya, 466-8550 Aichi Japan; 2 Department of Emergency Room General Medicine, Hyogo Prefectural Amagasaki General Medical Center, Higashinaniwa-Cho, Amagasaki, 660-8550 Hyogo Japan; 3 Institutes of Innovation for Future Society, Nagoya University, Hurou-Cho, Chikusa- Ku, Nagoya, 464-8601 Aichi Japan

**Keywords:** Hospitalization, Older adults, Energy intake, Undernutrition, Malnutrition, Appetite

## Abstract

**Background:**

Undernutrition is a prevalent issue among hospitalized older adults. Dietary intake is a major factor in nutritional status. Although insufficient dietary intake during hospitalization has been well documented, little research has focused on dietary intake at discharge, which may reflect the patient’s true dietary intake capacity. Furthermore, it would be desirable for energy intake to be estimated in a clinically feasible way.

**Objective:**

This study aimed to assess pre-discharge energy intake using routinely recorded dietary intake data, and to examine the association between pre-discharge energy intake and post-discharge mortality in older inpatients.

**Methods:**

A prospective cohort study was conducted in a geriatric ward. Energy intake in the 3 days prior to discharge was estimated using visually assessed dietary intake data recorded in medical records. The primary outcome was 3-month post-discharge mortality.

**Results:**

A total of 257 patients (mean age 84.7 years) was included. The mean pre-discharge energy intake was 1327 ± 315 kcal/day, with 74.7% of patients failing to meet recommended energy intake levels. Within 3 months post-discharge, 18 patients (7.0%) had died. Cox regression analysis revealed that higher pre-discharge energy intake was associated with lower post-discharge mortality (per 100 kcal/day, hazard ratio 0.75, 95% confidence interval 0.65–0.86), independent of other prognostic factors such as comorbidities and functional status.

**Conclusion:**

Many patients did not reach their recommended energy intake at discharge. Integrating routine monitoring of pre-discharge energy intake into discharge planning could trigger timely nutritional interventions and goal-of-care discussions, thereby improving post-discharge outcomes.

**Supplementary Information:**

The online version contains supplementary material available at 10.1007/s40520-025-03076-7.

## Introduction

Undernutrition is a common issue among hospitalized older adults, with an estimated prevalence of 20–50% [1]. In this population, undernutrition is associated with various adverse outcomes, including increased mortality, complications, and prolonged hospital stay [2]. One primary cause of undernutrition is insufficient dietary intake [3]. Previous studies have reported inadequate dietary intake during hospitalization in 30–90% of patients [4, 5, 6]. Inadequate energy intake during hospitalization is associated with undesirable outcomes, including in-hospital or post-discharge mortality [7, 8].

Hospitalized patients may experience reduced dietary intake for a variety of reasons, including loss of appetite due to diseases and fasting required for procedures and treatments. However, once a patient’s condition stabilizes, such as at the time of discharge, these factors are expected to have less influence. In other words, pre-discharge dietary intake may reflect the patient’s true dietary intake capacity. Although several studies have evaluated energy intake during hospitalization [7, 8], few have focused on energy intake at discharge. In one study involving patients hospitalized for heart failure (mean age 74 years), approximately half of the patients had an insufficient dietary intake at discharge, which was associated with an increased risk of adverse outcomes after discharge (composite of all-cause mortality and/or heart failure readmission within 1 year) [9]. Similar findings were reported in another study conducted in patients hospitalized for heart failure [10]. However, research on pre-discharge energy intake and its relationship to post-discharge outcomes has been limited to disease-specific populations, with no studies focusing on the general population of older inpatients. Older adults are hospitalized for a wide range of primary diagnoses, and in many hospitals they are preferentially admitted to general medical wards rather than disease- or organ-specific units. Consequently, clinical evidence derived exclusively from heart-failure cohorts cannot be uncritically extrapolated to the broader inpatient population. To address this gap, we conducted the present study in patients admitted to a geriatric ward, which routinely cares for older adults with a wide range of primary diagnoses.

In prior studies investigating the relationship between the energy intake and prognosis of hospitalized patients, dietary intake was assessed for research purposes, separately from routine clinical data [7, 8, 9, 10]. However, in clinical practice, dietary intake tends to be estimated visually, a method routinely utilized around the world [11]. Visual estimation of dietary intake has been shown to correlate well with the more accurate weighing method [12]. Using routinely collected dietary data has the potential to improve the generalizability of research findings.

The aim of this study was to assess energy intake at discharge using routinely recorded clinical data, and to examine the association between pre-discharge energy intake and post-discharge adverse outcomes in patients admitted to a geriatric ward.

## Methods

### Study design

This study analyzed data from a previously published prospective observational cohort study conducted in an acute geriatric unit of a university hospital [13]. The study protocol was approved by the Ethics Committee of Nagoya University Graduate School of Medicine (approval number 2019 − 0260) and conducted according to the principles of the Declaration of Helsinki and its later amendments. Written informed consent was obtained from all patients or a family member if necessary.

### Participants

The original cohort study included patients aged 65 years or older admitted to the Department of Geriatrics, Nagoya University Hospital from July 1, 2019 to May 31, 2023. Exclusion criteria for the original study were as follows: (1) discharged within 48 h; (2) inability to provide written informed consent; (3) life expectancy estimated to be less than 1 month at admission by the attending physician; (4) readmission within 3 months after discharge and enrolled in the study at the time of the previous hospitalization; (5) transferred from another hospital department; (6) and any other reason precluding participation, as determined by the attending physician. In the present study, we further excluded the following patients: those who died during hospitalization, were transferred to other departments, had not yet been discharged by the end of the study period, had been discharged for less than 3 months by the end of the study period, had a prognosis that was not known at 3 months after discharge, had received a peripheral or central intravenous infusion containing sugar before discharge, had missing energy intake, height or body weight data, or had missing data on the Mini Nutritional Assessment Short-Form (MNA-SF) [14] or basic activities of daily living (BADL).

### Data collection

Data were collected within the first 48 h of admission, at discharge, and 3 months after discharge.

#### At admission

The following data were obtained at the time of admission during patient and family interviews or from the electronic medical records: age, sex, height, weight, route of admission, pre-admission residence, and total number of medications. Patients’ functional capacity and cognitive function were assessed using the following: the Barthel Index [15] for BADL at baseline (2 weeks prior to admission); Lawton’s Scale [16] for instrumental activities of daily living at baseline (2 weeks prior to admission); and the Mini Mental State Examination [17] (within 7 days of admission) for cognition. In addition, the Charlson Comorbidity Index (CCI) [18] and Geriatric Depression Scale-15 [19] were completed, and nutritional status was assessed at admission using the MNA-SF.

#### At discharge

The following data were recorded at the time of discharge: days in hospital, weight at discharge, Barthel Index at discharge, and food intake in the 3 days prior to discharge (not including the day of discharge). A 3-day window was selected because (1) patients are considered clinically stable when discharge is anticipated, so dietary intake during this period best reflects their true intake capacity; (2) a 3-day average minimizes daily variation and provides a more reliable estimate than a single-day record; and (3) this approach has been adopted in a prior study [10].

#### At 3 months after discharge

Three months after discharge from the hospital, interviews were conducted by telephone with the patient or family members and the following information was collected: surviving or not, residence/place of care, falls or not, emergency room (ER) visits or not, and readmission or not.

### Energy intake Estimation

In Japan, nurses routinely assess food intake visually and record it in the medical records [20], and so we used such data to calculate each patient’s average food intake. Intake data for meals were recorded separately for staple and side dishes, each rated on an 11-point scale from 0 to 100% in 10% increments. Daily energy intake was estimated by multiplying the calories of the meal provided by the percent of the main meal and side dishes consumed. As an example, suppose on a given day that the patient consumed 40% of the staple food and 60% of the side dishes, and that overall they consumed (40% + 60%) / 2 = 50% of the total. If the meal provided was 1400 kcal/day, it was estimated that 1400 kcal/day × 50 / 100 = 700 kcal/day was consumed. The average of three meals (breakfast, lunch, and dinner) was calculated for both the staple and side dishes; however, in the case that any data were missing from the records for one meal, the daily average was estimated from the remaining two meals.

Visual estimation of energy intake by nursing staff has been shown to correlate well with weight-based food estimation of energy intake [12]. However, for non-solid foods, the correlation between the actual amount of food consumed and the nurse’s record is not clear [21], and thus may affect the results. Therefore, we counted the number of patients eating non-solid foods.

### Estimated energy needs

Estimated energy needs were based on the Dietary intake standards for Japanese people [22]. For inactive older adults, energy needs are estimated to be 2050 kcal/day for male and 1550 kcal/day for female aged 65 to 74, and 1800 kcal/day for male and 1400 kcal/day for female aged 75 and older.

### Outcome

The major outcome was all-cause mortality within 3 months of discharge. Secondary outcomes were falls, ER visits, and readmissions within 3 months of discharge. Observation was terminated at the end of the 3-month post-discharge interview.

### Statistical analysis

Data are presented as the mean ± standard deviation or median (interquartile range, 25th–75th percentile). Patients were divided into two groups based on energy intake, mean energy intake as the cutoff, and baseline characteristics were compared using the *t*-test, Mann–Whitney *U* test, or chi-square test. Furthermore, patients were divided into 4 groups by quartiles of energy intake, and differences in post-discharge 3-month mortality among 4 groups were evaluated using Cochran-Armitage test for trend.

Distributions of energy intake according to time to death within 3 months were evaluated using Kaplan-Meier survival curves. The survival curves are presented for 4 groups stratified by energy intake quartiles.

Hazard ratios were calculated using Cox regression analysis, with mortality within 3 months of discharge as the primary outcome. Covariates were age, sex, body mass index (BMI), energy intake, CCI, MNA-SF, BADL at discharge, and length of hospital stay (days). To evaluate model performance, the Akaike Information Criterion (AIC) and Harrell’s concordance index were calculated for the Cox proportional hazards models. A lower AIC indicates a better goodness of fit, while a higher concordance index suggests greater discriminative ability. To assess the impact of energy intake on model performance, AIC and concordance index values were also calculated for models excluding energy intake as a covariate. A sensitivity analysis was conducted excluding patients on non-solid foods.

Subanalyses included multiple logistic regression analysis with falls, ER visits, and rehospitalizations as dependent variables.

A *p*-value of < 0.05 was considered statistically significant. All statistical analysis was performed using IBM SPSS Statistics version 29.0.1.0 (171).

## Results

### Patient characteristics

**Fig. 1:**
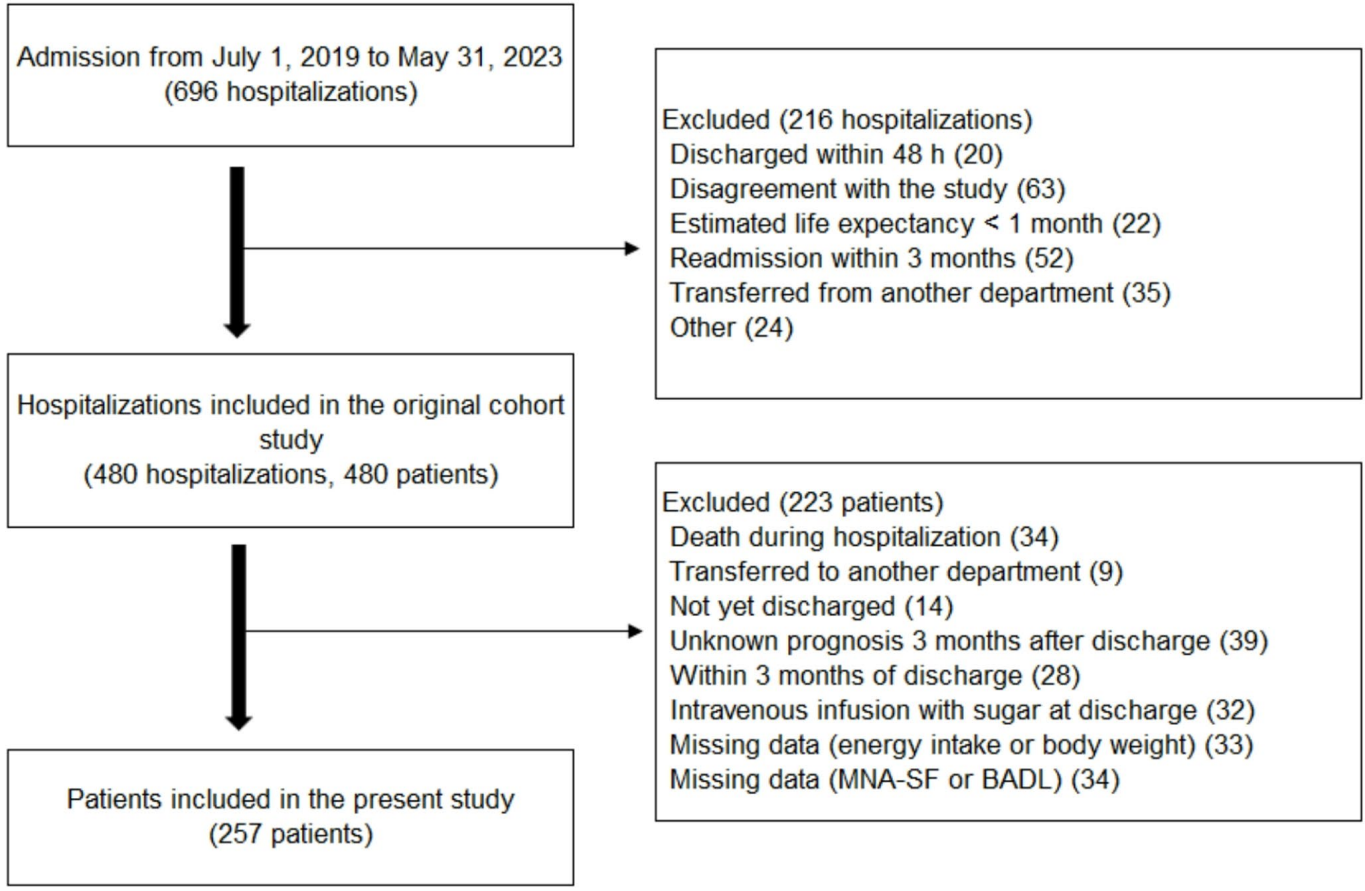
Flow diagram of patient selection

The original cohort study included 480 patients. After excluding 223 patients, 257 patients were included in the present analysis (Fig. 1).

The baseline characteristics are shown in Table 1. The mean age was 84.7 years, and 59.5% were female. The median length of hospital stay was 17 days. Two patients were on non-solid food. The mean energy intake prior to discharge was 1327 ± 315 kcal/day (28.9 ± 8.3 kcal/kg/day), and 192 patients (74.7%) did not reach the recommended energy intake. The low energy intake group was older, had a higher proportion of female, a higher risk of malnutrition on admission, lower ADL and lower body weight at discharge, and a higher mortality rate within 3 months of discharge.

**Table 1:** Baseline characteristics and outcomes of the study population

	All cases (*n* = 257)	Energy intake^a^		*p* ^b^
		Low (*n* = 94) (36.6%)	High (*n* = 163) (63.4%)	
Age, years	84.7 ± 5.8	86.0 ± 5.3	83.9 ± 5.9	0.004
Female sex	153 (59.5)	71 (75.5)	82 (50.3)	< 0.001
Emergency admission	165 (64.2)	71 (75.5)	94 (57.7)	0.004
Pre-admission residence				
Home	227 (88.3)	77 (81.9)	150 (92.0)	0.015
Nursing home	25 (9.7)	16 (17.0)	9 (5.5)	0.030
Other	5 (1.9)	1 (1.1)	4 (2.5)	0.66
MNA-SF^c^	9 (7–12)	8 (7–10)	10 (7–12)	< 0.001
BADL^d^ (at admission) (*n* = 255)	90 (65–100)	85 (55–95)	95 (80–100)	< 0.001
IADL^e^(*n* = 256)	5 (1–7)	3 (1–7)	6 (3–8)	< 0.001
MMSE^f^(*n* = 251)	22 (16–27)	21 (15–26)	24 (19–28)	< 0.001
GDS-15^g^(*n* = 231)	4 (2–8)	6 (3–9)	4 (2–8)	0.11
CCI^h^	2 (1–3)	2 (1–3)	2 (1–3)	0.92
Congestive heart failure	42 (16.3)	21 (22.3)	21 (12.9)	0.048
Stroke	61 (23.7)	19 (20.2)	42 (25.8)	0.31
Diabetes	73 (28.4)	22 (23.4)	51 (31.3)	0.18
Number of medications (at admission)	6 (4–9)	6 (3–9)	6 (4–9)	0.39
BADL (at discharge)	85 (55–100)	70 (45–90)	90 (80–100)	< 0.001
Body weight (at discharge), kg	47.7 ± 11.0	44.2 ± 9.6	50.7 ± 12.0	< 0.001
BMI, kg/m^2^	20.8 ± 4.1	20.2 ± 4.1	21.2 ± 4.0	0.07
Non-solid food	2 (0.8)	2 (2.1)	0 (0)	0.13
Energy intake, kcal/day	1327 ± 315	1006 ± 264	1513 ± 147	< 0.001
Energy intake per body weight, kcal/kg/day	28.9 ± 8.3	23.8 ± 7.7	31.6 ± 7.2	< 0.001
Energy intake above recommendation	65 (25.3)	0 (0)	65 (39.9)	< 0.001
Length of hospital stay, day	17 (11–28)	17 (11–31)	16 (11–25)	0.21
Outcomes				
Death within 3 months of discharge	18 (7.0)	14 (14.9)	4 (2.5)	< 0.001
Falls (*n* = 213)	33 (15.5)	9 (12.9)	24 (16.8)	0.46
ER visit (*n* = 217)	33 (15.2)	13 (18.1)	20 (13.8)	0.41
Readmission (*n* = 220)	49 (22.3)	17 (23.0)	32 (21.9)	0.86

Data are presented as the median (IQR), Mean ± SD, or no. (%)

^a^ Low is ≤ 1327 kcal/day, High is > 1327 kcal/day

^b^ Comparison between the two groups based on energy intake

^c^ MNA-SF ranges from 0 to 14 (a higher score indicates a lower risk of undernutrition)

^d^ BADL ranges from 0 to 100 (a higher score indicates better function)

^e^ IADL ranges from 0 to 8 (a higher score indicates better function)

^f^ MMSE ranges from 0 to 30 (a higher score indicates better function)

^g^ GDS-15 ranges from 0 to 15 (a higher score indicates more severe depression)

^h^ CCI ranges from 0 to 37 (a higher score indicates more severe comorbidities)

BADL: basic activities of daily living, BMI: body mass index, CCI: Charlson Comorbidity Index, GDS-15: Geriatric Depression Scale-15, IADL: instrumental activities of daily living, IQR: interquartile range, MMSE: Mini Mental State Examination, MNA-SF: Mini Nutritional Assessment short-form, SD: standard deviation

Table S1 shows the most common primary diseases among the patients. Compared with the 223 patients who were excluded from the study, those who were included were younger, had a higher MNA-SF score at admission, higher energy intake before discharge, and a higher Barthel Index at discharge (Table S2).

### Energy intake and 3-month post-discharge mortality

**Fig. 2:**
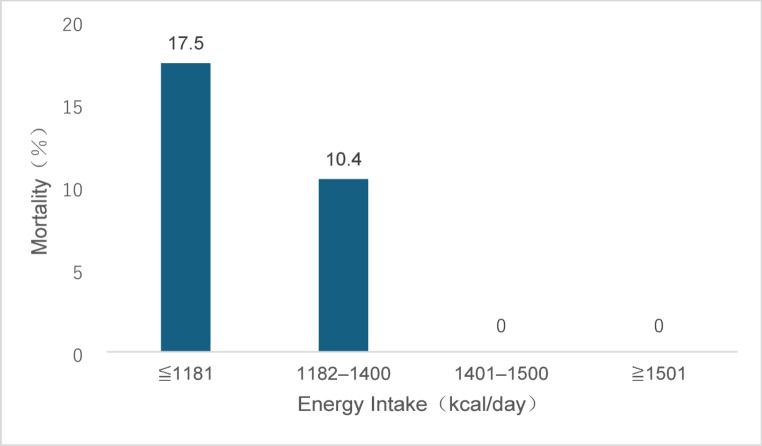
Three-month post-discharge mortality by quartile of energy intake Patients (*n* = 257) were stratified into 4 groups based on quartiles of energy intake in the 3 days prior to discharge (median 1400.0 kcal/day; interquartile range 1181.5–1500.0 kcal/day). The group with higher energy intake had a lower mortality rate (test for trend, *p* < 0.001)

Among the 257 patients, 18 (7.0%) died within 3 months of discharge. Patients with higher energy intake prior to discharge had lower post-discharge mortality rates (test for trend, *p* < 0.001) (Fig. 2).

**Fig. 3:**
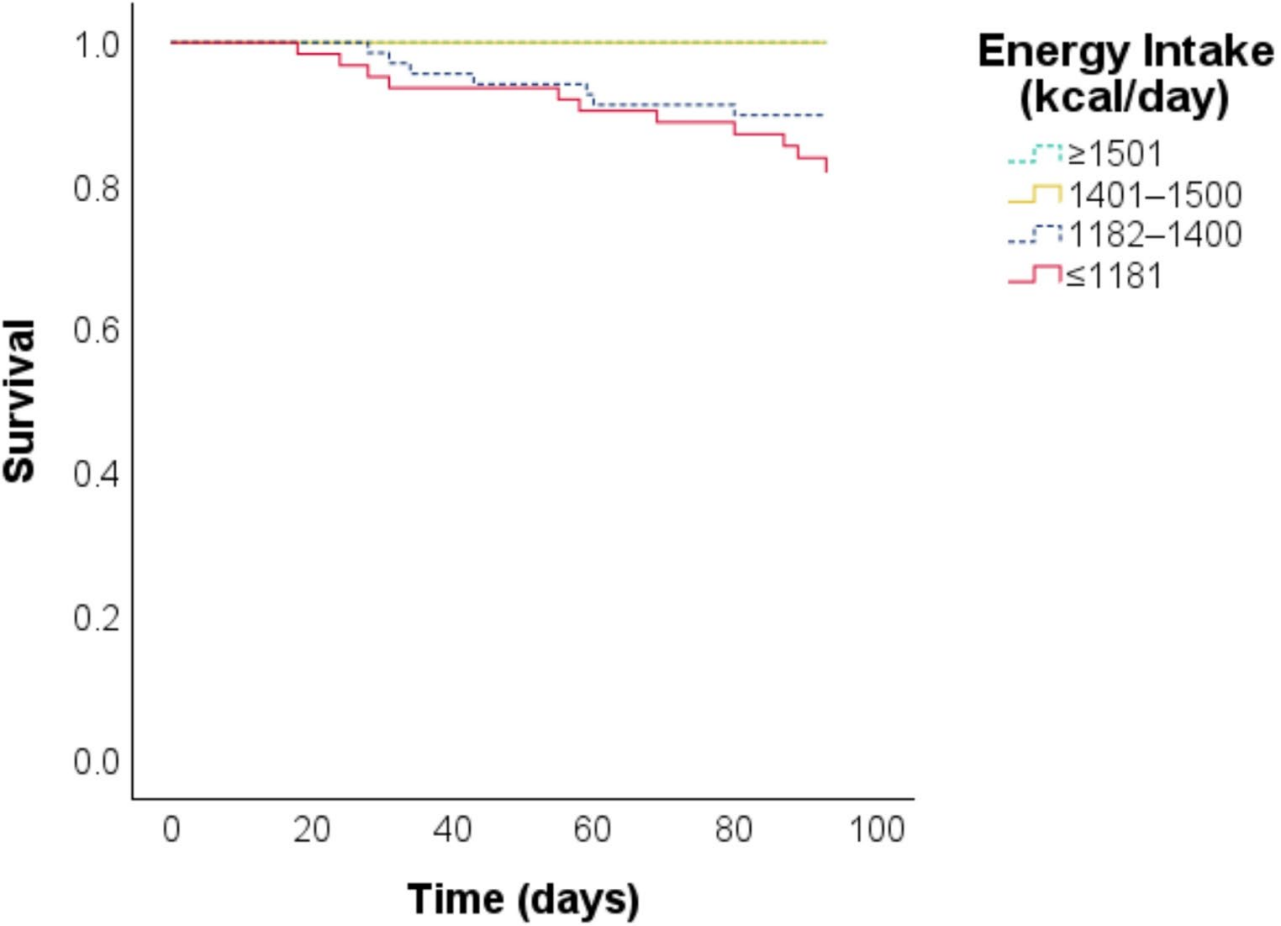
Kaplan-Meier curves for 3-month post-discharge survival by energy intake quartiles during the 3 days before discharge (*n* = 257; log-lank test, *p* < 0.001)

The Kaplan-Meier survival curves showed a clear separation between the two groups with higher energy intake and the two groups with lower energy intake (log-rank test, *p* < 0.001) (Fig. 3).

Cox regression analysis revealed that energy intake was associated with 3-month post-discharge mortality (per 100 kcal/day, OR 0.75; 95% CI: 0.65–0.86) independently of BMI, MNA-SF, CCI, BADL at discharge, and length of hospital stay (Table 2).

**Table 2:** Associations between energy intake and death within 3 months of discharge: results of Cox regression analysis

		HR	95%CI	*p*-value
model 1^a^	Age, years	1.06	0.96–1.16	0.24
	Female sex	0.42	0.14–1.25	0.12
	Energy intake, per 100 kcal/day	0.74	0.65–0.84	< 0.001
model 2^b^	Age, years	1.07	0.96–1.18	0.22
	Female sex	0.39	0.12–1.29	0.12
	Energy intake, per 100 kcal/day	0.75	0.65–0.86	< 0.001
	BMI	0.85	0.72–1.00	0.06
	MNA-SF	1.05	0.88–1.26	0.58
	CCI	1.34	1.07–1.66	0.009
	BADL (at discharge)	0.99	0.97–1.00	0.09
	Length of hospital stay, day	1.02	0.99–1.05	0.16

^a^ model 1: Covariates included age, sex, and energy intake

^b^ model 2: Covariates included age, sex, energy intake, BMI, MNA-SF, CCI, BADL, and length of hospital stay

See Table 1 footnotes for the range and meaning of each item.

BMI: body mass index, BADL: basic activities of daily living, CCI: Charlson Comorbidity Index, CI: confidence interval, MNA-SF: Mini Nutritional Assessment short-form, HR: hazard ratio

Table S3 presents the performance of each model. The AIC and concordance index for the model that included all variables (model 2) were 174.7 and 0.835, respectively. Models that included energy intake had a lower AIC and a higher concordance index compared to those without energy intake.

The sensitivity analysis revealed that excluding patients on non-solid foods (*n* = 2) did not impact the results (Table S4).

### Energy intake and other outcomes

There was no clear association between energy intake and rehospitalization, ER visits, or falls within 3 months after discharge (Table S5).

## Discussion

In this study conducted in a geriatric ward, many patients did not reach their recommended energy intake prior to discharge, according to routinely recorded dietary intake data. Energy intake was associated with 3-month post-discharge mortality, even after adjusting for known prognostic factors such as nutritional status on admission.

### Energy intake prior to discharge

A significant number of patients did not meet the recommended energy intake before discharge. The patients in this study had a relatively low mean BMI (20.8 kg/m^2^), raising concerns about further weight loss due to insufficient food intake.

A comparable study [10] investigated energy intake before discharge in patients with heart failure, reporting an energy intake of 1481 kcal/day (assessed visually by nurses) for 3 days before discharge. Their observed energy intake value was higher than that in the present study (1327 kcal/day). The discrepancy may be attributable to differences in patient age, given that our patients were older (mean age 84.7 vs. 78 years) and likely frailer. This might have led to longer recovery periods and some patients might have still been recovering at the time of discharge. Additionally, differences in underlying condition might have influenced energy intake. While Katano et al. focused on patients with heart failure, our study included individuals hospitalized for stroke, pneumonia, dementia, or appetite loss—conditions commonly associated with eating or swallowing difficulties.

Despite the lower absolute energy intake observed in our study, the energy intake per body weight was higher than that in the aforementioned study (28.9 vs. 27.0 kcal/kg/day) [10]. The lower body weight of our patients (47.7 vs. 51.6 kg) might have resulted in an overestimation of energy intake when expressed per kilogram of body weight, consistent with previously reported findings [23, 24].

Another study by Yoshida et al. [24] assessed food intake before discharge in patients with heart failure, using visual evaluations by nurses and researchers. However, energy intake was not reported, limiting direct comparisons.

### Energy intake prior to discharge and death within 3 months of discharge

Pre-discharge energy intake, derived from routine clinical data, was associated with 3-month post-discharge mortality, independent of other prognostic factors such as nutritional measures and ADLs.

Previous studies have also reported an association between low energy intake before discharge and worse post-discharge outcomes [9, 10]. However, these studies focused on patients with heart failure, and food intake was assessed for research purposes. In contrast, the present study included patients with various diseases, and used routinely recorded intake data, improving the generalizability of the findings.

There are several possible explanations for the mechanism underlying the observed association between low pre-discharge energy intake and post-discharge mortality. First, insufficient dietary intake may contribute to increased mortality. Although we did not evaluate post-discharge food intake, patients with inadequate intakes prior to discharge may have continued to consume insufficient amounts at home [25]. Although controlled caloric restriction has shown benefits in middle-aged or obese older adults [26, 27], its effects on frail, undernourished older adults remain unclear. Excessive caloric restriction has been shown to be harmful even in younger individuals [28]. In the present study, the mean energy intake in the low energy intake group was 1006 kcal/day, which is much less than the recommendation, and most of the patients who died within 3 months of discharge were in this group. Second, dietary intake may be an indicator of short life expectancy; in other words, dietary intake may be one of the indicators of a patient’s general health status. This is supported by a previous study, which reported that dietary intake decreased rapidly for several months before death in frail older adults [29]. In the present study, we found that energy intake was associated with post-discharge mortality, independent of physical function (BADL) and comorbidities (CCI). Dietary intake may reflect factors influencing general health status that were not captured in the present analyses.

### Implications for clinical practice and further research

These findings underscore the need to monitor and address low energy intake, even in patients deemed ready for discharge. One potential approach to integrate these findings into daily clinical practice is to implement a system that triggers an alert when energy intake is insufficient at the start of discharge planning. Such a system could facilitate early interventions, including post-discharge nutritional support, before the patient is discharged.

In nutrition care, energy intake is a fundamental parameter. The energy intake assessment method used in this study has high potential for practical application in clinical settings, and may contribute to the standardization of the nutrition care process. Implementing automated calculations of energy intake based on meal provision and actual consumption into electronic medical records would further facilitate those approaches.

Another implication is that energy intake may serve as a simple predictor of mortality, even when estimated using routinely recorded data. When energy intake remains low despite clinical improvement, this finding could trigger an urgent discussion about goals of care.

A randomized controlled trial by Schuetz et al. [6] demonstrated that nutritional support during hospitalization improved clinical outcomes, including mortality. However, the study population was relatively young (mean age 72.6 years). Further research is needed to ascertain whether nutritional interventions can enhance dietary intake and clinical outcomes in older, frail, and undernourished populations.

### Limitations

This study has several limitations. First, because this was a single-site prospective observational study, selection bias may have influenced the results. Ongoing multicenter data collection aims to address this limitation. Second, although the analyses included physical function (BADL) and comorbidities (CCI), disease severity was not considered, and this might have impacted both pre-discharge intake and post-discharge mortality. Third, this study did not assess the relationship between macronutrient intake and mortality, because energy intake was estimated using routinely recorded, visually assessed data. However, visual assessment methods are simple and applicable to other facilities. Finally, anthropometric parameters such as BMI vary according to race; therefore, the results of this study may not be generalizable to other ethnic groups.

## Conclusions

This prospective cohort study conducted in a geriatric ward revealed that many patients did not achieve adequate energy intake at discharge. Pre-discharge energy intake, based on routinely recorded dietary data, was independently associated with 3-month post-discharge mortality. Further studies in diverse settings are warranted.

## Electronic supplementary material

Below is the link to the electronic supplementary material.

Supplementary Material 1

## Data Availability

No datasets were generated or analysed during the current study.
