# Supplementary material for: Pre-discharge energy intake and post-discharge mortality in acutely hospitalized older adults
Source: Aging Clin Exp Res. 2025 May 26;37(1):170. doi: 10.1007/s40520-025-03076-7 (PMC12106510; doi:10.1007/s40520-025-03076-7)
Supplement: Supplementary file 1 — Supplementary Material 1 [file 40520_2025_3076_MOESM1_ESM.docx]

| Table. S1 Most common primary reason for admission | | | |
| --- | --- | --- | --- |
|  |  |  |  |
| Rank | Major diagnostic category | n | %^a^ |
| 1 | Stroke | 29 | 11.3 |
| 2 | Urinary tract infection | 28 | 10.9 |
| 3 | Loss of appetite | 24 | 9.3 |
| 4 | Pneumonia | 18 | 7.0 |
| 4 | Dementia | 18 | 7.0 |
| 6 | Collagen disease | 12 | 4.7 |
| 6 | Sepsis^b^ | 12 | 4.7 |
| 8 | Gastrointestinal disorder | 10 | 3.9 |
| 8 | Electrolyte disturbance | 10 | 3.9 |
| 10 | Heart failure | 9 | 3.5 |
|  | ^a^ Proportion of cases in relation to total number of admissions (n=257). | | |
|  | ^b^ Sepsis took precedence as the disease. For example, sepsis as a result of urinary tract infection was classified as sepsis. | | |

| **Table S2.** Comparisons of baseline characteristics between included and excluded patients | | | |  |
| --- | --- | --- | --- | --- |
|  |  |  |  |  |
|  | Included (n=257) | Excluded (n=223) | p |  |
| Age, years | 84.7 ±5.8 | 85.8 ±5.8 | 0.040 |  |
| Female sex | 153 (59.5) | 127 (57.0) | 0.57 |  |
| Emergency admission | 165 (64.2) | 178 (79.8) | <0.001 |  |
| Pre-admission residence |  |  |  |  |
| Home | 227 (88.3) | 185 (83.0) | 0.09 |  |
| Nursing home | 25 (9.7) | 31 (13.9) | 0.12 |  |
| Other | 5 (1.9) | 7 (3.1) | 0.40 |  |
| MNA-SF | 9 (7–12) | 8 (5–11) | 0.008 |  |
| BADL (at admission) | 90 (65–100) | 75 (35–100) | <0.001 |  |
| IADL | 5 (1–7) | 2 (0–6) | <0.001 |  |
| MMSE | 22 (16–27) | 18 (8–24) | <0.001 |  |
| GDS-15 | 4 (2–8) | 6 (3–9) | 0.13 |  |
| CCI | 2 (1–3) | 2 (1–4) | 0.033 |  |
| Congestive heart failure | 42 (16.3) | 44 (19.7) | 0.31 |  |
| Stroke | 61 (23.7) | 55 (24.7) | 0.85 |  |
| Diabetes | 73 (28.4) | 61 (27.4) | 0.77 |  |
| Number of medications (at admission) | 6 (4–9) | 6 (3–9) | 0.72 |  |
| BADL (at discharge) | 85 (55–100) | 55 (10–90) | <0.001 |  |
| Body weight (at discharge), kg | 47.7 ±11.0 | 47.0 ±10.3 | 0.54 |  |
| BMI, kg/m^2^ | 20.8 ±4.1 | 20.3 ±3.8 | 0.19 |  |
| Energy intake, kcal/day | 1327 ±315 | 1082 ±349 | <0.001 |  |
| Energy intake per body weight, kcal/kg/day | 28.9 ±8.3 | 23.1 ±8.2 | <0.001 |  |
| Length of hospital stay, day | 17 (11–28) | 19 (11–33) | 0.16 |  |
|  |  |  |  |  |
| See Table 1 footnotes for the range and meaning of each item. | | | | |
|  |  |  |  |  |
| BMI: body mass index, BADL: basic activities of daily living, CCI: Charlson Comorbidity Index, GDS-15: Geriatric Depression Scale-15, IADL: instrumental activities of daily living, MMSE: Mini Mental State Examination, MNA-SF: Mini Nutritional Assessment short-form | | | | |

| **Table S3.** Comparison of models for mortality within 3-month post-discharge with and without energy intake as a covariate: results of Cox proportional hazards regression analyses | | | |
| --- | --- | --- | --- |
|  | | | |
| Model | Variables | AIC | C-index |
| model 1 | age + sex + Energy intake | 180.9 | 0.822 |
| model 2 | age + sex + BMI + MNA-SF + CCI + BADL (at discharge) + LOS + Energy intake | 174.7 | 0.835 |
| model 3 | age + sex | 199.1 | 0.606 |
| model 4 | age + sex + BMI + MNA-SF + CCI + BADL (at discharge) + LOS | 188.6 | 0.776 |
|  |  |  |  |
| AIC: Akaike Information Criterion, BMI: body mass index, BADL: basic activities of daily living, CCI: Charlson Comorbidity Index, CI: confidence interval, LOS: length of stay, MNA-SF: Mini Nutritional Assessment short-form, | | | |

| **Table S4.** Results of sensitivity analyses excluding patients on non-solid food: association between energy intake excluding non-solid food and death within 3 months of hospital discharge (Cox regression analysis) (n = 255) | | | | |
| --- | --- | --- | --- | --- |
|  |  |  |  |  |
|  |  | HR | 95%CI | p-value |
| model 1^a^ | Age, years | 1.03 | 0.94–1.14 | 0.49 |
|  | Female sex | 0.37 | 0.12–1.13 | 0.08 |
|  | Energy intake, per 100 kcal/day | 0.73 | 0.64–0.83 | <0.001 |
|  |  |  |  |  |
| model 2^b^ | Age, years | 1.05 | 0.94–1.17 | 0.40 |
|  | Female sex | 0.40 | 0.12–1.40 | 0.15 |
|  | Energy intake, per 100 kcal/day | 0.76 | 0.65–0.88 | <0.001 |
|  | BMI | 0.85 | 0.71–1.01 | 0.06 |
|  | MNA-SF | 1.04 | 0.87–1.24 | 0.69 |
|  | CCI | 1.40 | 1.11–1.77 | 0.005 |
|  | BADL (at discharge) | 0.98 | 0.97–1.00 | 0.07 |
|  | Length of hospital stay, day | 1.01 | 0.98–1.05 | 0.48 |
|  |  |  |  |  |
| ^a^ model 1: Covariates included age, sex, and energy intake | | | | |
| ^b^ model 2: Covariates included age, sex, energy intake, BMI, MNA-SF, CCI, BADL, and length of hospital stay | | | | |
|  |  |  |  |  |
| See Table 1 footnotes for the range and meaning of each item. | | | | |
| BMI: body mass index, BADL: basic activities of daily living, CCI: Charlson Comorbidity Index, CI: confidence interval, MNA-SF: Mini Nutritional Assessment short-form, HR: hazard ratio | | | | |

| **Table S5.** Multiple logistic regression analysis of the association between energy intake and each outcome | | | | | | | | | | | | |
| --- | --- | --- | --- | --- | --- | --- | --- | --- | --- | --- | --- | --- |
|  |  |  |  |  |  |  |  |  |  |  |  |  |
|  |  | Outcome | | | | | | | | | | |
|  |  | Falls | | |  | ER visit | | |  | Readmission | | |
|  |  | OR | 95％CI | p |  | OR | 95％CI | p |  | OR | 95％CI | p |
| model 1^a^ | Age | 1.01 | 0.94–1.07 | 0.86 |  | 1.01 | 0.94–1.08 | 0.88 |  | 0.99 | 0.93–1.05 | 0.70 |
|  | Female | 1.20 | 0.54–2.65 | 0.66 |  | 0.64 | 0.29–1.42 | 0.27 |  | 0.55 | 0.28–1.09 | 0.09 |
|  | Energy intake  (per 100 kcal/day) | 1.10 | 0.95–1.28 | 0.21 |  | 0.91 | 0.79–1.04 | 0.17 |  | 0.93 | 0.83–1.05 | 0.26 |
| model 2^b^ | Age | 1 | 0.93–1.07 | 0.91 |  | 1.00 | 0.93–1.07 | 0.98 |  | 0.98 | 0.92–1.04 | 0.53 |
|  | Female | 1.01 | 0.43–2.36 | 0.98 |  | 0.65 | 0.28–1.51 | 0.32 |  | 0.51 | 0.25–1.05 | 0.07 |
|  | Energy intake  (per 100 kcal/day) | 1.16 | 0.99–1.37 | 0.07 |  | 0.93 | 0.81–1.08 | 0.35 |  | 0.96 | 0.85–1.09 | 0.54 |
|  | BMI | 0.98 | 0.87–1.10 | 0.73 |  | 1.02 | 0.91–1.14 | 0.79 |  | 0.99 | 0.90–1.10 | 0.87 |
|  | MNA-SF | 0.96 | 0.83–1.12 | 0.60 |  | 1.09 | 0.94–1.28 | 0.25 |  | 1.00 | 0.88–1.13 | 0.98 |
|  | CCI | 0.97 | 0.76–1.25 | 0.83 |  | 0.99 | 0.78–1.26 | 0.95 |  | 1.05 | 0.86–1.27 | 0.66 |
|  | BADL (at discharge) | 0.99 | 0.98–1.01 | 0.21 |  | 0.98 | 0.97–1.00 | 0.038 |  | 0.99 | 0.98–1.01 | 0.25 |
|  | Length of hospital stay | 1.01 | 0.99–1.04 | 0.25 |  | 0.99 | 0.96–1.02 | 0.33 |  | 1.01 | 0.99–1.04 | 0.21 |
|  |  |  |  |  |  |  |  |  |  |  |  |  |
| ^a^ model 1: Covariates included age, sex, and energy intake | | | | | | | | | | | | |
| ^b^ model 2: Covariates included age, sex, energy intake, BMI, MNA-SF, CCI, BADL, and length of hospital stay | | | | | | | | | | | | |
|  |  |  |  |  |  |  |  |  |  |  |  |  |
| CI: confidence interval, ER: emergency room, OR: odds ratio | | | | | | | | | | | | |
